# Fibroma osificante periférico. Una serie de casos

**DOI:** 10.21142/2523-2754-1303-2025-256

**Published:** 2025-08-31

**Authors:** Mariangela Domínguez Iafaioli, Alessandra Domínguez Iafaioli, Victoria Nogueira Colmenares, Jesús Pérez Cautela, Ligia Pérez Castro, Rafael Muñoz Morales

**Affiliations:** 1 Universidad Jose Antonio Paez. Valencia, Venezuela. mdomingueziafaioli@gmail.com, aledomiaf10@gmail.com, victorianatalicnogueira@gmail.com Universidad José Antonio Páez Universidad Jose Antonio Paez Valencia Venezuela mdomingueziafaioli@gmail.com aledomiaf10@gmail.com victorianatalicnogueira@gmail.com; 2 Hospital Universitario Dr. Angel Larralde. Valencia, Venezuela. perezcautela@gmail.com Hospital Universitario Dr. Angel Larralde Valencia Venezuela perezcautela@gmail.com; 3 Universidad del Zulia. Maracaibo, Venezuela ligiaperezcastro@gmail.com Universidad del Zulia Universidad del Zulia Maracaibo Venezuela ligiaperezcastro@gmail.com; 4 Ciudad Hospitalaria Dr. Enrique Tejera. Valencia, Venezuela. Ramm339@gmail.com Ciudad Hospitalaria Dr. Enrique Tejera Valencia Venezuela Ramm339@gmail.com

**Keywords:** fibroma osificante periférico, lesiones reactivas, escisión quirúrgica, recurrencia, peripheral ossifying fibroma, reactive lesions, surgical excision, recurrence

## Abstract

**Objetivo:**

Reportar una serie de tres casos diagnosticados con fibroma osificante periférico.

**Materiales y métodos:**

: Se presentaron tres casos clínicos de pacientes con lesiones exofíticas a las que se les realizó una biopsia escisional, con desbridamiento del hueso y, en casos específicos, la exodoncia de dientes asociados.

**Resultados:**

Los tres casos fueron diagnosticados como fibromas osificantes periféricos, tras la determinación de hallazgos histopatológicos consistentes con el diagnóstico, como la presencia de tejido fibroso, depósitos óseos y material calcificado. Tras un año de seguimiento, no se observó recurrencia en ninguno de los pacientes.

**Conclusiones:**

Estos casos destacan la importancia de una extirpación quirúrgica adecuada, que incluya márgenes de seguridad, junto con un seguimiento posoperatorio riguroso y el control de irritantes locales, a fin de prevenir la recurrencia de la lesión y garantizar la integridad funcional y estética de los tejidos afectados.

## SUMMARY

**Objective:**

To report a series of three cases diagnosed with peripheral ossifying fibroma.

**Materials and Methods:**

Three clinical cases are presented of patients with exophytic lesions who underwent excisional biopsy with bone debridement and, in specific cases, extraction of associated teeth.

**Results:**

the three cases were diagnosed as peripheral ossifying fibromas, subsequent to the determination of histopathological findings consistent with the diagnosis, such as the presence of fibrous tissue, bone deposits and calcified material. After 1 year of follow-up, no recurrence was observed in any of the patients.

**Conclusions:**

these cases highlight the importance of adequate surgical removal, including safety margins, along with rigorous postoperative follow-up and control of local irritants; In order to prevent recurrence of the injury, and ensuring the functional and aesthetic integrity of the affected tissues.

## INTRODUCCIÓN

El fibroma osificante periférico (FOP) es un crecimiento reactivo que representa aproximadamente el 3% de todos los tumores de la cavidad bucal y el 9,6% de todas las lesiones gingivales ^1^. Se localiza principalmente en la región maxilar anterior y presenta una incidencia significativa entre la tercera y cuarta décadas de vida; asimismo, afecta con mayor frecuencia al sexo femenino. El origen del FOP es controversial, si bien las teorías actuales sugieren que la irritación crónica y el traumatismo son agentes etiológicos significativos, ya que se considera que se desarrolla como una respuesta inflamatoria exagerada por parte de las células del tejido conectivo del ligamento periodontal ante estímulos desencadenantes ^2^^-^^4^.

Clínicamente, el fibroma osificante periférico se presenta como una lesión exofítica que puede ser pediculada o sésil. Su color varía entre rosado y rojo, y su tamaño generalmente no excede los dos centímetros ^2^^,^^5^. Radiográficamente, en algunos casos, es posible observar focos radiopacos asociados dependiendo de la cantidad de tejido calcificado presente ^4^^,^^5^.

El diagnóstico del FOP es exclusivamente histopatológico. Este fibroma se caracteriza por estar constituido por tejido conectivo fibroso altamente celular, con células mesenquimatosas fusiformes y ovoides, así como depósitos variables de hueso maduro, hueso inmaduro y calcificación distrófica. El tratamiento consiste en la resección local, con márgenes periféricos y profundos que incluyan el periodonto y periostio. Asimismo, es fundamental la eliminación de los factores etiológicos locales y establecer controles posoperatorios para monitorear posibles recidivas ^4^.

El objetivo del presente artículo es reportar una serie de tres casos diagnosticados con fibroma osificante periférico.

## MATERIALES Y MÉTODOS

Los tres casos clínicos reportados corresponden a pacientes atendidos en la Clínica de Cirugía Bucal de la Universidad José Antonio Páez, en el estado Carabobo, en Venezuela. Estos pacientes acudieron debido a la presencia de lesiones exofíticas en la cavidad bucal, las cuales fueron extirpadas y diagnosticadas como fibromas osificantes periféricos (FOP). Se realizó el seguimiento durante un año, período en el cual no se reportaron recidivas. A cada paciente se le proporcionó información tanto verbal como escrita, y se le solicitó firmar un consentimiento informado tras una explicación detallada de la naturaleza de la lesión y los pasos a seguir para su tratamiento. Los pacientes autorizaron el registro fotográfico para su divulgación científica. Esta investigación se adhiere a las directrices éticas de la Declaración de Helsinki ^6^.

## CASOS CLÍNICOS

### CASO 1

**Figura 1:**
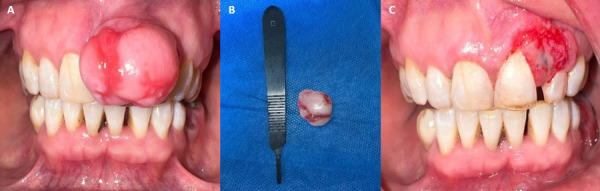
A) Fotografía intrabucal preoperatoria: lesión exofítica que emerge desde la región gingival anterior izquierda del maxilar. B) Vista macroscópica de la lesión. C) Posoperatorio 10 días después.

Se presenta el caso de paciente de sexo femenino, de 57 años, sin antecedentes médicos relevantes, quien acudió tras presentar aumento de volumen progresivo de 10 meses de evolución en la región maxilar anterior. La lesión, indolora y exofítica, medía aproximadamente 2 x 0,7 cm, presentaba un color similar al de la mucosa adyacente y discretas zonas eritematosas (Figura 1-A). Durante la evaluación, se identificaron irritantes locales como biofilm y cálculo dental. 

No se evidenciaron cambios significativos a nivel radiográfico luego de evaluar las reconstrucciones multiplanares de la tomografía del haz cónico. Se planteó como otro diagnóstico diferencial al granuloma piogénico. Se llevó a cabo una biopsia escisional con márgenes de seguridad de 2 mm. Posteriormente, se realizó el lavado de la zona con solución fisiológica al 0,9% y se optó por promover una cicatrización por segunda intención (Figuras 1B y 1C). El análisis histopatológico reveló fragmentos tisulares cubiertos por epitelio plano estratificado con acantosis moderada, infiltrado inflamatorio linfocitario focal e islotes irregulares de hueso compacto maduro y áreas de osificación, lo que confirmó el diagnóstico de fibroma osificante periférico. Durante el seguimiento posoperatorio, que se extendió hasta un año, no se evidenciaron indicios de la recidiva de la lesión.

### CASO 2

**Figura 2:**
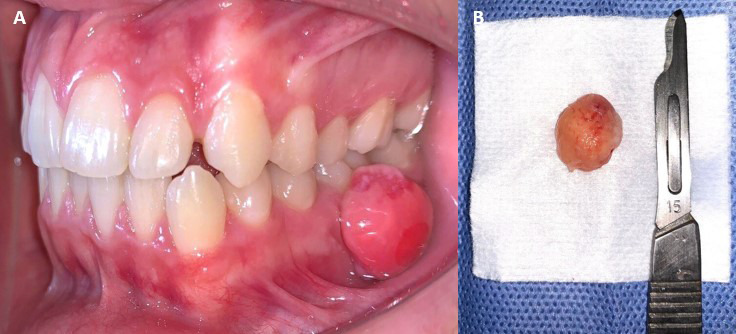
A) Vista intrabucal preoperatoria que muestra una lesión exofítica y pediculada, localizada en la región gingival mandibular izquierda, la cual compromete la superficie vestibular del OD 36. B) Lesión extirpada.

Se presenta el caso de un paciente de sexo masculino de 17 años, quien refirió inicio de enfermedad actual tras notar un aumento de volumen intrabucal de crecimiento progresivo, con 7 meses de evolución, en la región gingival mandibular posterior izquierda, específicamente en la superficie de la encía vestibular del OD 36. Clínicamente, se observó una lesión exofítica, de forma redondeada, pediculada y de color rosado similar a la mucosa adyacente. La lesión era indolora y medía aproximadamente 1,4 x 1,0 cm. Se identificaron focos de irritantes locales, como biofilm (Figura 2A). Luego de la evaluación imagenológica, mediante una tomografía computarizada de haz cónico, no se evidenciaron hallazgos asociados con la entidad. Se plantearon, juntamente con el FOP, otros dos posibles diagnósticos diferenciales: el granuloma piógeno y granuloma periférico de células gigantes.

**Figura 3:**
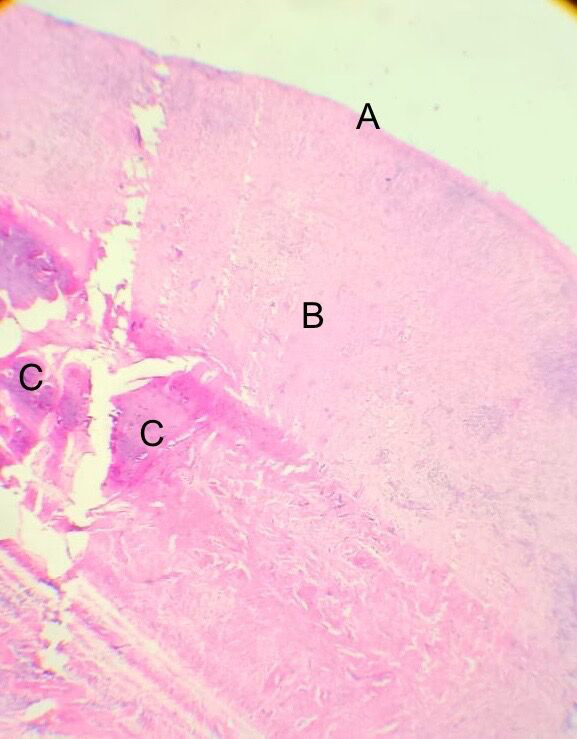
Fotomicrografía de tinción H&E (40X): A) superficie epitelial ulcerada. B) estroma de tejido conectivo fibroso. C) calcificaciones.

La resolución quirúrgica se efectuó mediante la biopsia escisional, en donde se la lesión fue abordada desde su inserción pediculada, respetando 2 mm de tejido sano circundante. La zona fue cureteada y, posteriormente, se realizó un lavado del lecho quirúrgico con solución fisiológica al 0,9% (Figura 2B). Al examen microscópico, se observó un epitelio escamoso estratificado, con áreas hiperplásicas y otras ulceradas, sobre un tejido conectivo fibroso que presentaba abundantes calcificaciones, además de un moderado infiltrado inflamatorio mononuclear. Estos hallazgos fueron compatibles con un fibroma osificante periférico (Figura 3). Durante el seguimiento posoperatorio, efectuado durante un año, no se evidenciaron recurrencias.

### CASO 3

**Figura 4:**
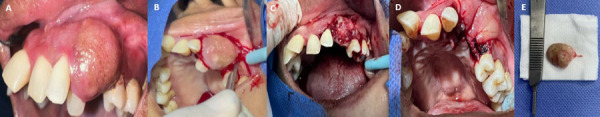
A) Vista intrabucal preoperatoria. Lesión localizada en la región gingival maxilar izquierda, con desplazamiento del OD 23. B) Incisión. C) Lecho posquirúrgico. D) Colgajo de avance vestibular para cierre de defecto y síntesis de los tejidos. E) Vista macroscópica de la lesión.

Se describe el caso de paciente del sexo femenino de 44 años, con antecedente médico de hipertensión arterial, quien refirió el inicio de la enfermedad actual tras un aumento de volumen intrabucal, de 5 años de evolución, en la región maxilar anterior izquierda. La lesión exofítica era de forma ovalada, color violáceo y base sésil, y medía aproximadamente 2,5 x 2,5 cm de diámetro. Emergía desde la encía vestibular y se extendía hacia la superficie palatina, lo que causaba el desplazamiento del OD 23. Se identificaron irritantes locales asociados a biofilm y una condición periodontal severa con movilidad dentaria (Figura 4A), por lo que, antes de la intervención, se ameritó el control de irritantes presentes. No se evidenciaron cambios imagenológicos en la tomografía computarizada de haz cónico. Con base en los hallazgos, se plantearon los diagnósticos diferenciales de fibroma osificante periférico y granuloma piógeno.

Se realizó una biopsia escisional respetando los márgenes quirúrgicos (Figuras 4B y 4C), y se efectuó la odontectomía del OD 23. Luego, se llevó a cabo un curetaje exhaustivo de la zona. Para el cierre del defecto, se procedió al levantamiento de un colgajo de avance vestibular (FIGURAS 4D y 4E) y la posterior síntesis de los tejidos. El estudio histopatológico reveló una lesión revestida por epitelio plano estratificado queratinizado, con haces de fibras colágenas y focos densos de infiltrado inflamatorio crónico, así como la presencia de depósitos irregulares de tejido óseo calcificado, lo que determinó un diagnóstico definitivo de fibroma osificante periférico. Se realizó una monitorización posoperatoria durante un año, en la cual no se observó la recurrencia de la lesión.

## DISCUSIÓN

El fibroma osificante periférico es una proliferación inflamatoria no neoplásica y reactiva que ocurre como parte de una respuesta inflamatoria de las células del ligamento periodontal ante estímulos asociados a traumatismos o irritantes locales ^4^^,^^5^.

Se revisaron series de casos de FOP informados en los últimos cinco años (Tabla 1). Los hallazgos indican un predominio femenino constante en el 60% de los casos. Se observó que la ubicación de las lesiones se restringió exclusivamente a zonas gingivales, siendo la región mandibular anterior la mayormente afectada (40%). En el contexto de los tres casos reportados en esta investigación, es posible establecer patrones y discrepancias interesantes. En primer lugar, la tendencia del sexo femenino en nuestros casos es consistente con la observada en la literatura ^2^^,^^6^^-^^11^.

**Table:** 

Autor	Sexo y edad	Tiempo de evolución	Ubicación	Tamaño	Características clínicas	Características radiográficas	Características histopatológicas
Nasser *et al*. ^12^.	F-25 años	5 años	Mandibular anterior izquierda	4,1 cm	Color rojo-rosado, pediculada, superficie ulcerada	Desplazamiento dentario, pérdida ósea	Infiltrado inflamatorio crónico, fibroblastos fusiformes blandos, tejido óseo.
Godinho *et al*. ^13^.	F-53 años	10 años	Maxilar posterior izquierda	5 x 4,5 cm	Color rosado, liso, pediculada	Imagen radiopaca, pérdida ósea	Infiltrado inflamatorio mononuclear, material osteoide.
Pereira *et al*. ^14^.	F-26 años	2 meses	Mandibular anterior	1,2 x 0,8 cm	Color rojo-rosado, pediculada	-	Infiltrado inflamatorio crónico y vasos sanguíneos. Material osteoide y hueso reticulado.
Cansu *et al*. ^15^.	M-34 años	1 año	Maxilar anterior izquierda	2,1 x 1,9 cm	Color rojo-rosado, pediculada	Imagen radiopaca	Infiltrado inflamatorio crónico, áreas mineralizadas y calcificación distrófica.
El Gaouzi *et al*. ^1^.	F-42 años	2 años	Mandibular anterior y posterior izquierda	6 x 4 cm	Color rojo-rosado, sésil	Hiperdensidad y desplazamiento dentario, sin destrucción ósea	Proliferación fibroósea. Trabéculas óseas bordeadas por un borde de osteoblastos.
Seo *et al*. ^11^.	M-63 años	3 meses	Maxilar posterior izquierda	5 x 2,5 cm	Color rojo, sésil, ulcerada	Imagen radiopaca, pérdida ósea	Estroma de tejido conectivo fibroso. Material similar al cemento.
Dare *et al*. ^2^.	F-18 años	1 mes	Mandibular anterior	1 x 0,8 cm	Color rosado, sésil	-	Estroma de tejido conectivo fibrocelular. Áreas de calcificación distrófica en el tejido conectivo adyacente
Pandey *et al*. ^10^.	M-33 años	1 semana	Maxilar anterior	0,7 x 0,6 cm	Color rojo-rosado, pediculada	Imagen radiopaca, pérdida ósea	Estroma de tejido conectivo fibroso y trabéculas irregulares de hueso, osteoide y calcificaciones distróficas.
Xavier *et al*. ^9^.	F-50 años	5 meses	Mandibular posterior izquierda	2,1 x 1,5 cm	Color rosado, sésil	Desplazamiento dentario	Estroma de tejido conectivo fibroso con áreas de osificación y áreas con depósitos de cemento.
Takagi *et al*. ^16^	M-68 años	3 meses	Maxilar posterior derecha	6 x 3,6 cm	Color rojo-rosado, pediculada	Pérdida ósea	Componente fibroso con osificación y calcificación similares a cemento

La media de edad fue de 41,8 años en los casos resumidos en la tabla 1; en nuestros casos se exhibe un rango más amplio. Notoriamente, el segundo caso presenta a un paciente masculino de 17 años, que es significativamente más joven que la media reportada. La predilección por la región anterior del maxilar del primer y tercer caso discrepa de la tendencia observada en los casos reportados en los últimos cinco años; sin embargo, coinciden con los hallazgos encontrados en el estudio retrospectivo de Cavalcante *et al*. ^4^, quienes evaluaron 270 FOP y establecieron la región gingival del maxilar anterior como la mayormente afectada. 

La media asociada a la evolución de estas lesiones (tabla 1) fue de 1,92 años, entre 2 semanas y 10 años. Con base en esto, se han categorizado algunas condiciones asociadas a la presencia de los FOP de mayor tamaño: desplazamientos dentarios y movilidad, interferencia oclusal, incompetencia labial, dificultad para hablar y asimetría facial ^3^^,^^4^^,^^8^^-^^16^.

El tamaño promedio obtenido, considerando la dimensión más grande proporcionada, fue de 3,32 cm (tabla 1), lo que refleja una dimensión mayor en comparación con los datos de nuestra investigación ^1^^,^^2^^,^^10^^-^^15^, en donde la lesión con mayor dimensión midió 2,5 cm, lo que se corresponde con el tercer caso, respectivamente. Los hallazgos clínicos lo definen como una lesión exofítica pediculada o sésil, y con un color que varía entre rojo y rosado. Estas características exhiben similitudes clínicas comparables con otras lesiones que conforman sus diagnósticos diferenciales; como la hiperplasia fibrosa, el granuloma piógeno, el granuloma periférico de células gigantes y el fibroma odontogénico periférico ^1^^,^^4^^,^^8^^,^^12^^,^^14^^,^^17^.

Radiográficamente, el FOP puede no presentar alteraciones apreciables. Sin embargo, según el grado de mineralización, ciertos pacientes pueden mostrar variaciones en la radiodensidad dentro de la lesión, como en la variabilidad observada en la tabla 1. Por esta razón, se recomienda un enfoque de tratamiento exhaustivo, dado que la tasa de recurrencia es alta, aproximadamente del 20%. Este enfoque debe incluir la eliminación completa de la lesión, el desbridamiento del hueso y la exodoncia de dientes asociados ^1^^,^^3^^,^^4^^,^^14^. Particularmente, en nuestros casos, no se evidenciaron hallazgos a nivel imagenológico y, durante el periodo de seguimiento de un año, no se observó la recurrencia de las lesiones. Los datos histopatológicos reportados (tabla 1) incluyen la presencia de tejido conectivo fibroso con contenido variable de fibroblastos, miofibroblastos y colágeno, proliferación endotelial de escasa a abundante y diferentes tipos de material mineralizado, como cemento, óseo y calcificaciones distróficas. 

Se han documentado casos inusuales de FOP con dimensiones considerables (≥ 6 cm), como el descrito por Takagi *et al*. ^16^, en el que se evidenció una gran destrucción ósea del alvéolo. En este caso, los antecedentes de tabaquismo del paciente, la rápida progresión de la lesión, los hallazgos clínicos y la ausencia de características radiográficas que sugirieran focos de calcificación llevaron a los autores a plantear inicialmente la posibilidad de que se tratara de una lesión maligna. Esto pone en evidencia la capacidad del FOP para simular otras entidades, que difieren además en la terapéutica indicada para su resolución y ameritan una intervención oportuna; por tanto, su distinción histopatológica es un aspecto elemental.

Por otra parte, Godinho *et al*. ^13^ reportaron un caso atípico de un FOP que, tres años antes, había sido diagnosticado como un granuloma piógeno. Posteriormente, en función de los cambios histopatológicos observados, se realizó el diagnóstico de FOP, evidenciado por el desarrollo de material calcificado, maduración fibrosa y una disminución del contenido vascular en la lesión. Esto sugiere que algunos granulomas piógenos pueden evolucionar hacia un FOP; sin embargo, esto no lo restringe como un diagnóstico primario, por lo que se requieren más estudios al respecto para la compresión del comportamiento de la lesión y esclarecer la posible correlación entre estas dos entidades.

## CONCLUSIONES

El fibroma osificante periférico es una lesión reactiva de naturaleza inflamatoria, cuyas características clínicas pueden asemejarse a otras entidades dentro de esta categoría. Radiográficamente, se suelen observar radiopacidades asociadas al contenido calcificado de la lesión; sin embargo, en los casos presentados, no se identificaron. Por lo tanto, el diagnóstico definitivo requiere un estudio histopatológico que confirme la naturaleza de esta entidad. El manejo del FOP implica la extirpación quirúrgica completa con márgenes de seguridad, pero es crucial un seguimiento posoperatorio riguroso y una monitorización periódica para garantizar la ausencia de irritantes locales y el control de hábitos traumáticos que puedan predisponer su recidiva.

## Financiamiento:

No se recibió ningún tipo de financiamiento.
